# Genomic Effects Associated With Response to Placebo Treatment in a Randomized Trial of Irritable Bowel Syndrome

**DOI:** 10.3389/fpain.2021.775386

**Published:** 2022-01-04

**Authors:** Rui-Sheng Wang, Anthony J. Lembo, Ted J. Kaptchuk, Vivian Cheng, Judy Nee, Johanna Iturrino, Meenakshi Rao, Joseph Loscalzo, Jocelyn A. Silvester, Kathryn T. Hall

**Affiliations:** ^1^Department of Medicine, Brigham Women's Hospital, Boston, MA, United States; ^2^Department of Medicine, Harvard Medical School, Boston, MA, United States; ^3^Division of Gastroenterology, Beth Israel Deaconess Medical Center, Boston, MA, United States; ^4^Program in Placebo Studies, Beth Israel Deaconess Medical Center, Boston, MA, United States; ^5^Department of General Medicine Primary Care, Beth Israel Deaconess Medical Center, Boston, MA, United States; ^6^Department of Pediatrics, Boston Children's Hospital, Boston, MA, United States; ^7^Department of Pediatrics, Harvard Medical School, Boston, MA, United States; ^8^Celiac Disease Program, Boston Children's Hospital, Boston, MA, United States

**Keywords:** irritable bowel syndrome (IBS), placebos, catechol-O-methyltransferase (*COMT*), randomized control trial (RCT), gene expression

## Abstract

**Background and Aims:** Irritable bowel syndrome (IBS), a functional pain disorder of gut-brain interactions, is characterized by a high placebo response in randomized clinical trials (RCTs). Catechol-O-methyltransferase (*COMT*) rs4680, which encodes high-activity (val) or low-activity (met) enzyme variants, was previously associated with placebo response to sham-acupuncture in an IBS RCT. Examining *COMT* effects and identifying novel genomic factors that influence response to placebo pills is critical to identifying underlying mechanisms and predicting and managing placebos in RCTs.

**Methods:** Participants with IBS (*N* = 188) were randomized to three placebo-related interventions, namely, double-blind placebo (DBP), open-label placebo (OLP), or simply trial enrollment without placebo treatment [no placebo (i.e., no pill) treatment control (NPC)], for 6 weeks. *COMT* rs4680, gene-set, and genome-wide suggestive (*p* < 10^−5^) loci effects on irritable bowel symptom severity score (IBS-SSS) across all participants were examined.

**Results:** Participants with IBS homozygous for rs4680 met (met/met) had the greatest improvement across all arms, with significantly greater improvement compared to val/val in DBP (beta (SE), −89.4 (42.3); *p* = 0.04). Twelve genome-wide suggestive loci formed a gene regulatory network highly connected to *EGR1*, a transcription factor involved in placebo-related processes of learning, memory, and response to stress and reward. *EGR1* gene expression in peripheral blood mononuclear cells (PBMC) was significantly reduced at the endpoint across all treatment arms (log fold-change, −0.15; *p* = 0.02). Gene-set enrichment analysis returned three genome-wide significant ontology terms (GO:0032968, GO:0070934, and GO:0070937) linked to transcription regulation and GO:0003918 associated with DNA topoisomerase regulation.

**Conclusion:** These results suggest common molecular mechanisms in response to varying forms of placebo that may inform personalized IBS treatment and placebo response prediction.

**Clinical Trial Registration:** ClinicalTrials.gov, Identifier: NCT0280224.

## Introduction

Irritable bowel syndrome (IBS) is a highly prevalent disorder of the gut-brain interaction, characterized by abdominal pain and altered bowel function. Early life events, including psychological trauma and environmental exposures, such as gastrointestinal infections, increase susceptibility to IBS, and psychological stress frequently exacerbates symptoms. The use of double-blind placebo (DBP) controls in randomized clinical trials (RCT) is associated with high placebo response rates (average 40%) among participants with IBS. Recently, our group completed a 6-week RCT in IBS comparing DBP, open-label placebo (OLP), and simply enrolling in a trial with the patient-researcher engagement but no placebo (i.e., no pill) treatment control (NPC) (1). More than half of the participants in each placebo treatment arm had a >50-point improvement in the primary outcome IBS-symptom severity score (IBS-SSS). Participants randomized to DBP and OLP had similar improvement in IBS symptoms, and both had significantly greater improvement compared with NPC. Understanding the mechanisms underlying response to placebo treatment is critically important to managing placebo effects in IBS clinical care, RCT design, and drug development.

Neurological changes in response to placebo treatment have been mapped to specific brain regions implicated in reward salience, pain, and emotional processing. In the prefrontal cortex (PFC), activation of dopaminergic signaling pathways has been observed in models of placebo response in depression and Parkinson's disease (2). A key regulator of dopamine turnover in the PFC is catechol-O-methyltransferase (*COMT*), an enzyme that metabolizes endogenous catechol-containing neurotransmitters and hormones, including dopamine, norepinephrine, epinephrine, and catechol estrogen. The most studied single nucleotide polymorphism (SNP) in *COMT*, rs4680, encodes a G-to-A transversion, resulting in a valine (val)-to-methionine (met) substitution, and a three-to four-fold reduction in enzymatic activity (3, 4). In a previous randomized trial of placebo treatments in IBS, we reported the association of genetic variation at *COMT* rs4680 with placebo response to single-blinded sham acupuncture augmented with a warm-caring clinical interaction (5).

Response to placebo treatments is a complex phenotype likely influenced by multiple genomic factors in addition to genetic variation in *COMT*. However, large sample size is required to have adequate power to discern the small genomic effects typically observed in a genome-wide association study (GWAS). Hence, we combined the DBP, OLP, and NPC treatment arms, assuming that placebo-related effects would be present and contribute to response in each of the three treatment arms. Because this study was not well-powered to conduct a GWAS, we used gene-set analysis, which aggregates genome-wide association data into pathways and functions, to achieve the power required to identify significant biologically relevant effects.

To broaden our understanding of how genomic variation influences placebo response in IBS, here we examine candidate *COMT* rs4680 and genome-wide effects using gene-set and transcription network analysis across participants in our recently completed IBS RCT of three placebo treatments (i.e., DBP, OLP, and NPC) (1).

## Materials and Methods

### Study Design

Effects of open-label vs. double-blind treatment in IBS was a clinical trial that randomized IBS participants to one of three placebo treatments: DBP, OLP, or NPC (1). A small number of participants were randomized to a fourth arm [double-blind peppermint oil (DBM)] to allow for the DBP treatment arm. Because peppermint oil (6) is considered an active treatment, participants in this treatment arm were not included in the present analysis. Full details of the trial participants, design, and results have been previously published (7). Briefly, 340 IBS participants were randomized to one of the treatment arms for 6 weeks; 242 participants completed the study and had baseline and 6-week IBS-SSS, 188 of whom were randomized to one of the three placebo treatment arms (DBP, OLP, and NPC), consented to genetic analysis, and were successfully genotyped. The dual aims of the parent study were to compare OLP to NPC, and OLP to DBP. All participants attended in-person study visits at baseline, and at weeks 3 and 6, in which they met with a study clinician and completed the questionnaires. Blood for genotyping was drawn at the first visit. Blood for transcription analysis using RNA sequencing was drawn at baseline and 6 weeks.

### Ethics Approval Statement

This study and the parent trial were conducted according to the criteria set by the Declaration of Helsinki. All participants provided informed consent, and the study was approved by the ethics review board at Beth Israel Deaconess Medical Center under protocol 2015P000282.

### Outcome Measures

Outcome assessments were performed by blinded research assistants. OLP and NPC participants were not blinded; participants assigned to DBP or DBM were told they enrolled for a double-blind RCT but were not informed of their randomized treatment assignment. The primary outcome was changed in the irritable bowel symptom severity scale (IBS-SSS). IBS-SSS is a validated five-item questionnaire used to assess IBS symptoms and severity of the disease consisting of pain severity, pain frequency, bowel distension, satisfaction with bowel habits, and quality of life (6). Each item is scored on a scale of 1–100, and, thus, the maximum possible composite IBS-SSS score is 500. Higher scores are associated with more severe symptoms; the primary outcome, change in IBS-SSS, was determined as:


$$\begin{array}{l} \left(IBS-SSS\;at\;baseline\right)\;-\left(IBS-SSS\;at\;6\;weeks\right) \end{array}$$

Generally, in pharmaceutical RCTs, the time course of placebo responses for functional pain illnesses follows the time trajectory of the drugs (7). In IBS, even at a 1-week placebo, the drug effects are evident (8). In long term IBS drug RCTs (i.e., 26 weeks), placebo responses continue as long as the drug effect, and if there is any reduction in placebo effects, it matches with what happens with the drug (9). We chose 6 weeks as a primary endpoint measure because previous studies suggested that 6 weeks is a reasonable time frame to detect placebo and peppermint effects, and subsequent studies have confirmed this assumption.

### Power Calculations

In a previous IBS trial (5), the mean (SD) in IBS-SSS score change by *COMT* rs4680 genotype with sham acupuncture was 87.4 (85.3) for met/met; 69.2 (70.5) for val/met; and 36.3 (74.4) for val/val. Thus, we estimated that we had >80% power to detect a difference between the two homozygous groups with an *n* of 188.

### Genotyping and Gene Expression

Additional information regarding genotyping on the Infinium Global Screening Array v2.0 (Illumina, San Diego, and Calif) and RNA-seq (Differential Gene Expression Analysis) performed at Admera Health (Plainfield NJ) on RNA extracted from human blood using PAXgene Blood RNA kit (Qiagen, Hilden, and Germany) at baseline and 6 weeks is available in the Supplementary Material.

### Candidate Gene, Gene-Set, and GWAS Analysis

For the GWAS, the following model was utilized:


$$\begin{array}{l} \text{IBS-SSS}\;change\;\sim \;SNP\;+\;age\;+\;sex\;+\;treatment\;arm\\ \;+\;5\;principal\;components\;\left(PCs\right) \end{array}$$

The top five principle components were used to correct for genetic heterogeneity across different races/ethnic groups. Principle components analysis (PCA) was performed on the whole genome SNP data using PLINK (10). In GWAS of quantitative change, the baseline measure has been shown to bias the effect of variants on treatment response; therefore, we did not include baseline IBS-SSS as a covariate in the model (11).

For this analysis, plink (10) was used to determine the effects of gene dosage for SNPs with a frequency >0.05. SNPs were considered to be genome-wide suggestive or significant if they were associated at thresholds of *p* < 10^−5^and *p* < 5.0 × 10^−8^, respectively. The GWAS output was cleaned using EasyQC with standard settings. Manhattan and QQ plots were generated with R package qqman.

We used FUMA (http://fuma.ctglab.nl/) to generate gene-based tests and extract functional annotations for genome-suggestive loci (*p* < 10^−5^). Summary statistics from the FUMA GWAS analysis were used to run multimarker analysis of GenoMic annotation (MAGMA) (12). In the gene-set analysis, MAGMA tests if the results from the gene-based analysis point to the involvement of specific pathways; *p* < 4.6 x 10^−6^ is considered to be significant. Analysis of transcription factor networks was performed using NetworkAnalyst 3.0 (13).

## Results

### Demographics and Baseline Measures of Participants

**Figure 1:**
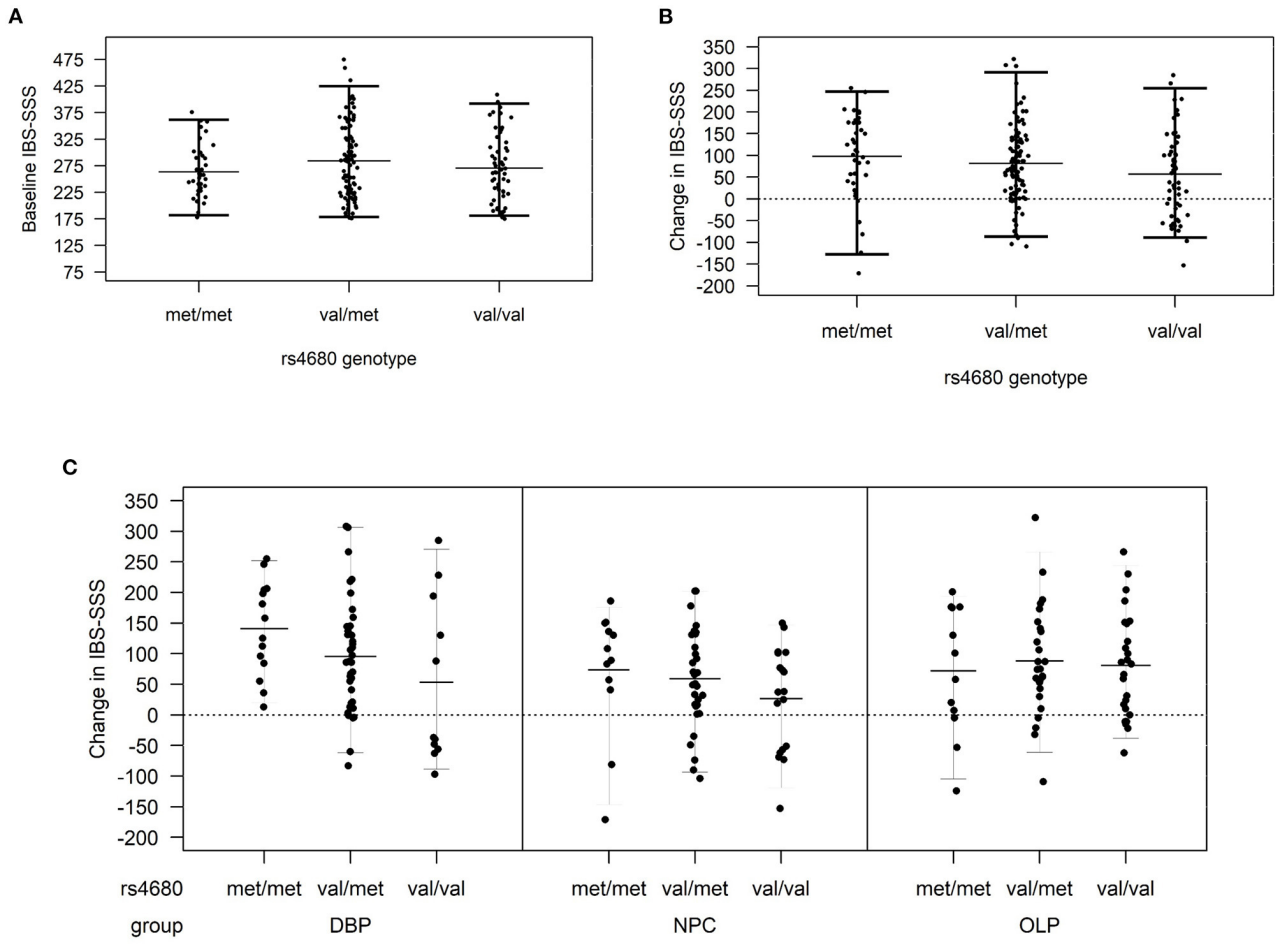
**(A)** Baseline irritable bowel symptom severity score (IBS-SSS) by *COMT* genotype across all treatment arms, **(B)** change in IBS-SSS by *COMT* rs4680 genotype overall, and **(C)** by treatment arm (DBP, double-blind; OLP, open-label and NPC, no-pill control).

This study examined 188 participants with IBS enrolled in a RCT (1, 14) who were randomized to DBP (*N* = 63), OLP (*N* = 63), or NPC (*N* = 62). The distribution of demographic and baseline clinical characteristics did not vary by randomized treatment allocation (Table 1). The average age of participants was 42.1 ± 18.2 years, 73% were women, and a majority (85%) self-reported their race as white. The distribution of *COMT* rs4680 was in Hardy-Weinberg equilibrium (*p* = 0.93). At baseline, IBS-SSS did not vary by treatment arm (Table 1) or by *COMT* rs4680 genotype across all arms combined (Figure 1A).

**Table 1:** Demographics, baseline characteristics, and COMT rs4680 distribution by treatment arm.

	**Double-blind**	**Open-label**	**No-pill**
	**placebo (DBP)**	**placebo (OLP)**	**control (NPC)**
N	63	63	62
Age, mean (SD)	43.2 (19.8)	43.2(17.3)	40.1 (17.6)
Female, *n* (%)	45 (70)	48 (76)	44 (71)
White, *n* (%)	54 (86)	53 (84)	52 (84)
IBS-SSS, mean (SD)	283.1 (69.8)	282.7 (57.4)	261.8 (66.2)
***COMT*** **rs4680**			
met/met (%)	14 (22)	12 (19)	12 (19)
val/met (%)	38 (60)	26 (41)	32 (52)
val/val (%)	11 (18)	25 (40)	18 (29)

### COMT Association With Change in IBS-SSS

In *COMT* rs4680 gene dosage models of change in IBS-SSS from baseline to 6 weeks, increasing the number of met alleles was associated with a greater reduction in IBS symptom severity (beta (SE), −22.3 (10.0), *p* = 0.027) such that participants homozygous for the low activity met allele (met/met) had the greatest placebo response across all participants in the three treatment arms combined (Figure 1B).

In gene dosage models stratified by treatment arm, the largest difference by *COMT* genotype was observed in the DBP. Specifically, met/met participants had the largest improvement with DBP (140.6 ± 77.2), val/met participants were intermediate (95.10 ± 92.6), and val/val (53.1 ± 136.5) participants had the smallest change (beta (SE), −90.1 (40.6); *p* = 0.04) (Figure 1C). In the NPC arm, the pattern was similar to DBP, but the change in IBS-SSS was lower in magnitude and the differences by *COMT* genotype were non-significant (beta (SE), −27.1 (16.7); *p* = 0.11). There was no difference by *COMT* genotype in OLP (*p* = 0.79).

Stratification by sex revealed a similar pattern of *COMT* rs4680 effects across all three treatment arms in women, such that met/met women had the greatest improvement (109.9 ± 96.5) and val/val women the least improvement (67.4 ± 97.4; Supplementary Figure 1). This pattern was observed in men in the DBP and NPC, but not in men randomized to OLP.

### Genome-Wide Association Analysis

**Figure 2:**
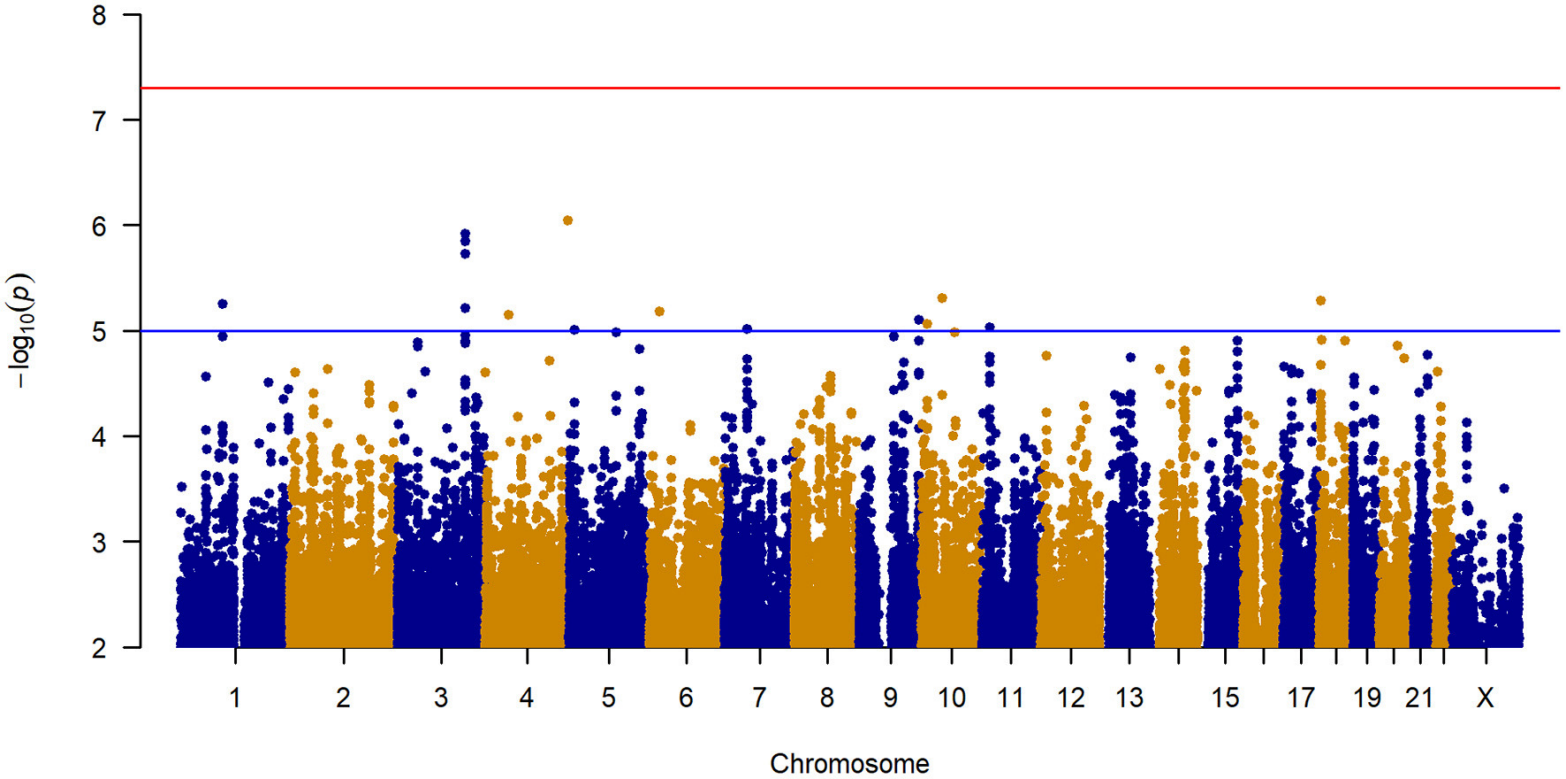
Manhattan plot of GWAS of change in IBS-SSS among 188 IBS patients controlling for age, sex, treatment arm, and the first five principal components for genetic ancestry.

No inflation of data was observed in the GWAS of change in IBS-SSS from baseline to 6 weeks across all treatment arms (Figure 2 and Supplementary Figure 2). The 12 loci associated with a change in IBS-SSS at the genome-wide suggestive level (set at *p* < 10^−5^) are described in Table 2. Seven loci mapped to introns, one to an exonic region in a non-coding RNA, and the rest were located in intergenic regions. Several loci had links to neuronal and gastrointestinal function and one, *NAV2* (neuron navigator 2), had links to placebo response (15). *NAV2* is critical to vagus nerve development (16), is associated with gut microbiome composition (17), and was previously associated with placebo response in asthma (15). *CTNND2* is associated with severe pain (18) and anxiety (19). *LINC02006*, a non-coding RNA, is associated with gut microbiota (20), serotonin levels (21), and infantile hypertrophic pyloric stenosis (22). Other genome-wide suggestive loci were linked to genes involved in neuronal growth, connection, and signaling [*COBL* (23), *DCDC2* (24)*, PTBP2* (25), *CTNND2* (19), and *ZBTB14* (26)].

**Table 2:** Genome-wide suggestive loci associated with the change in IBS-SSS from baseline to 6 weeks in three placebo treatment arms combined.

**rsID**	**Location**	**MAF**	**Gene (nearest)**	***P-*** **value**	**Type**	**Description**	**TP53**	**EGR1**
rs6701417	1:97071826	0.24	(PTBP2)	5.60E-06	intergenic	This SNP maps upstream of and is an eQTL for PTPBP2.		
rs57519743	3:153297025	0.18	LINC02006/ LINC02877	1.87E-06	intronic	In GWAS LINC02006 was associated with gut microbiota, serotonin levels, infantile hypertrophic pyloric stenosis.		
rs28652757	4:53881711	0.23	SCFD2	7.17E-06	intronic	In GWAS sec1 family domain containing 2 was associated with testosterone levels and uterine fibroids.		
rs6815638	4:188225599	0.21	AC097652.1 (FAT1)	9.08E-07	non-coding RNA exonic	NA		
rs31947	5:11461390	0.07	CTNND2	9.85E-06	intronic	Catenin delta 2 plays a critical role in neuronal development and formation and maintenance of dendrites and synapses.		
rs62400400	6:24266331	0.12	DCDC2	6.59E-06	intronic	Doublecortin domain containing 2 - plays a role in neuronal migration and ciliogenesis.		
rs9649794	7:51649139	0.41	AC005999.2	9.75E-06	intergenic	Cordon-bleu WH2 repeat protein regulates neuronal morphogenesis and increases axon and dendrite branching. It is required for growth and assembly of brush border microvilli that maintain intestinal homeostasis.		
rs11244033	9:136079182	0.34	(OBP2B)	7.87E-06	intergenic	This SNP maps proximal to and is an eQTL for odorant binding protein 2B		
rs12266806	10:12978973	0.08	CCDC3	8.76E-06	intronic	Coiled-coil domain containing 3 is highly conserved secretory protein that represses TNF-alpha/NF-KB and regulates liver lipid metabolism.		
rs11259792	10:47691930	0.15	ANTXRL	4.93E-06	intronic	Anthrax toxin receptor-like—is associated with bipolar disorder		
rs11025279	11:19853393	0.34	NAV2	4.93E-06	intronic	Neuron navigator 2—may play a role in neuronal growth and migration, is associated with gut microbiome composition and was associated with placebo response in asthma		
rs142674057	18:5307474	0.08	(ZBTB14)	5.18E-06	intergenic	Zinc Finger And BTB Domain Containing 14—transcriptional activator of dopamine transporter (DAT) and IL-6.		

*Columns correspond to SNP name, chromosomal location, (MAF), gene symbol for gene or nearest gene in brackets, p-value, SNP type, description of the function of the protein. Gray shaded boxes indicate genes that contain transcription binding sites for TP53 or EGR1*.

### Gene-Set Analysis

**Figure 3:**
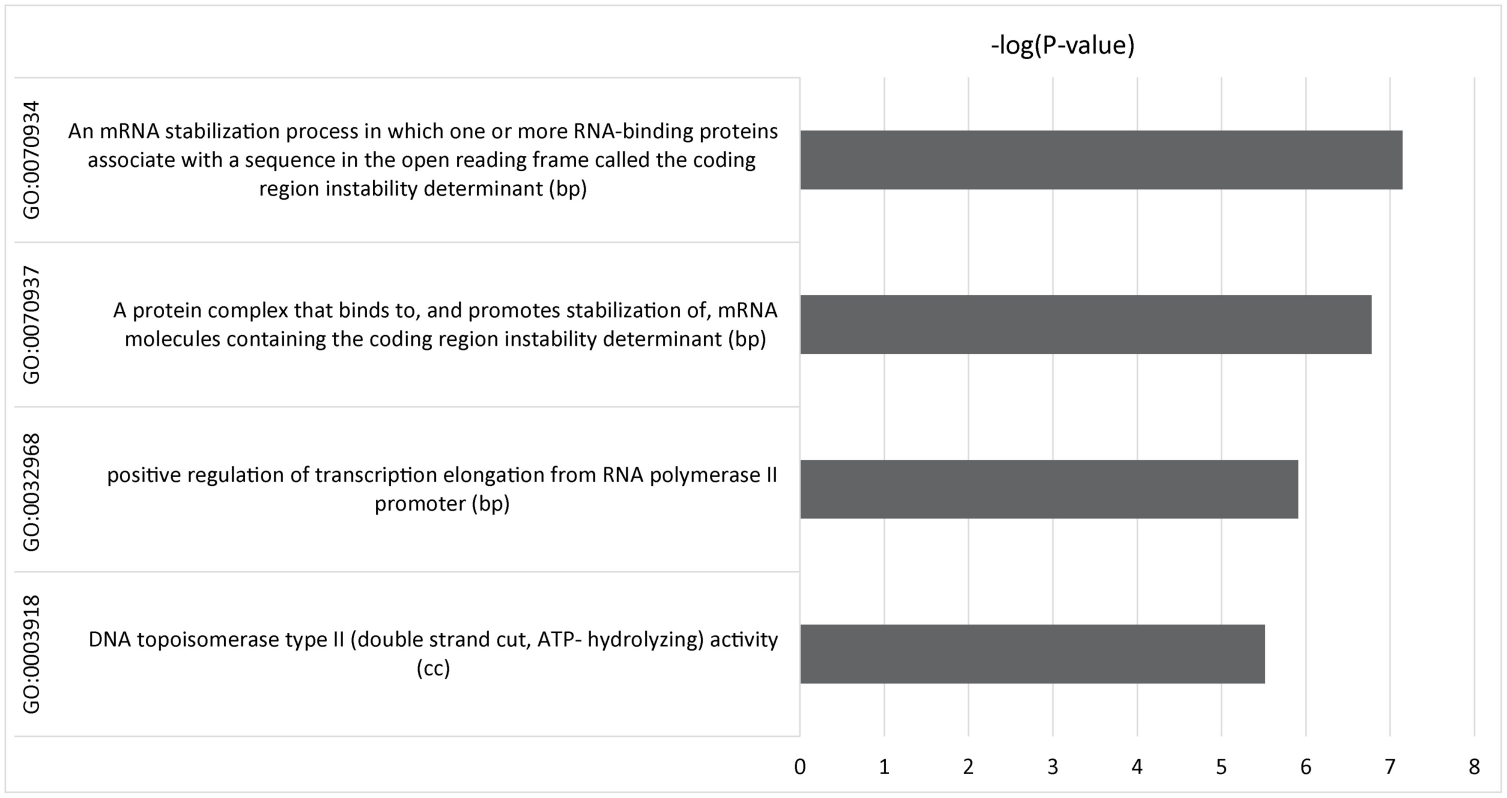
Multimarker analysis of GenoMic annotation (MAGMA) output of significant gene sets after gene set analysis of 15,488 tests was performed and Bonferroni corrected. Relative GO terms are biological processes (bp) and cellular components (cc).

We used gene-set enrichment analysis (12) to identify pathways or genes with common functions associated with IBS symptom improvement. Four gene ontology (GO) terms were identified that were genome-wide significant after Bonferroni correction (Figure 3 and Supplementary Table 1). Three pathways were involved in transcriptional regulation: GO:0032968, *p* = 1.23 x 10^−6^, which is involved in the regulation of transcription elongation from RNA polymerase II promoter; GO:0070937, *p* = 1.66 x 10^−7^, and the related GO:0070934, *p* = 7.14 x 10^−8^, which mediate stabilization of mRNA by RNA-binding proteins associated with the open reading frame (27); and GO:0003918, *p* = 3.06 x 10^−6^, which is associated with DNA topoisomerase activity (28).

### Gene Expression Network Analysis

**Figure 4:**
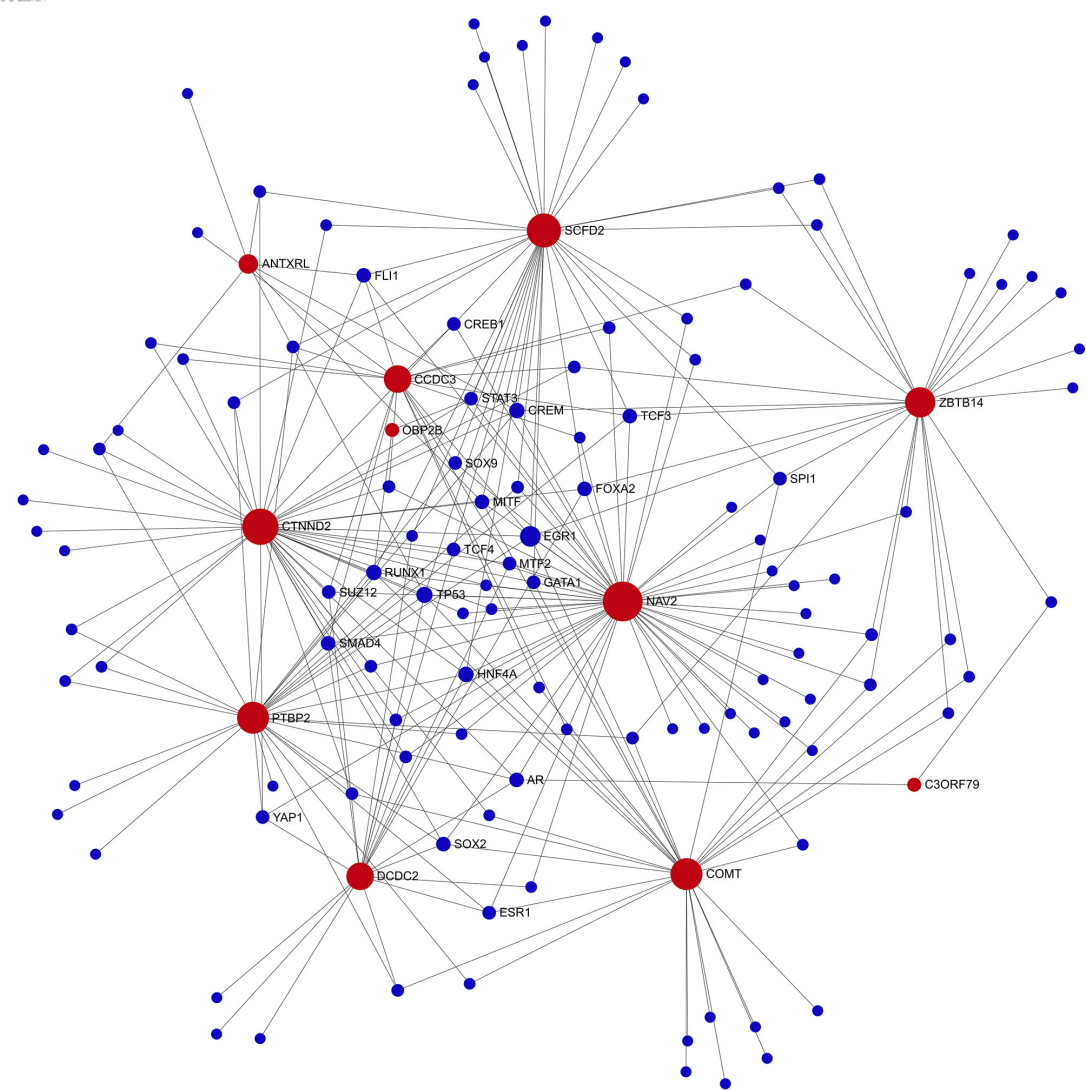
Transcription factor network diagram consisting of seed genes (11-top genome-wide suggestive (red) genes and *COMT*). Seed genes (red) with interaction partners (blue).

Gene regulatory network analysis of the genome-suggestive loci identified a transcription factor network that included 10/12 loci plus *COMT* (Figure 4). *EGR1* was the transcription factor with the highest degree (7) and betweenness centrality (407); *TP53* also had a degree of 7 (Supplementary Table 2). *EGR1* is rapidly induced by physiologic or emotional stress to upregulate transcription of a wide set of genes, including those involved in dopamine synthesis.

Comparison of transcript levels in peripheral blood samples from the IBS participants across all three treatment arms at baseline and 6-weeks indicated that *EGR1* gene expression was significantly reduced across all treatment arms (log fold-change −0.15; *p* = 0.02; *N* = 188). Changes in *TP53* gene expression were not significant (*p* > 0.05).

## Discussion

In this study, in a clinical trial of patients with IBS, randomized to three placebo-related interventions (DBP, OLP, and NPC), we found the effects of *COMT* rs4680 in response to placebo treatments. Particularly in DBP, met/met participants had a significantly greater improvement in IBS symptoms compared with participants who were met/val and val/val. Furthermore, assuming that placebo-related response would be present to varying degrees in each arm, we identified transcription regulation and *EGR1* gene expression as novel epigenetic processes that potentially influence response to placebo treatment in IBS.

The COMT enzyme metabolizes several hormones and neurotransmitters, including norepinephrine, dopamine, and catechol estrogen, which have been implicated in IBS pathophysiology, stress, and response to placebo treatments. The low-activity form of the COMT enzyme, encoded by a methionine (met) allele in the rs4680 genetic polymorphism, ostensibly results in higher levels of these COMT substrates. Notably, there was no difference in IBS-SSS by *COMT* rs4680 at baseline, so it is unlikely that the changes in response to placebo treatment observed in this study were attributed to regression to the mean. Across the three placebo treatment arms in this study, participants homozygous for the met allele (met/met) had significantly greater improvement in the primary outcome measure, change in IBS-SSS, compared to homozygotes for the high-activity form of the enzyme (val/val). In women, the direction of the *COMT* rs4680 effect was consistent across all three arms. Apart from the OLP arm, the direction of *COMT* effects in men was similar to this overall trend of met/met > val/val. However, with so few men with the met/met genotype enrolled in this trial, follow-up studies are needed to understand if there are sex-specific responses to OLP. Taken together, this study extends our previous finding that genetic variation in *COMT* differentially influences IBS symptom improvement in response to placebo treatment (5), in particular DBP, and suggests that the *COMT* rs4680 genetic variant may be useful in predicting, managing, and targeting placebo response in IBS trials and drug development.

As a complex phenotype, placebo treatment response in IBS is likely to be polygenic, with influence from many genetic loci each with small individual effects. However, identifying small genetic effects requires a large sample size to provide power to discern statistically significant effects. As expected in a study with a sample size having limited power, none of the loci in the GWAS reached genome-wide significance. Gene-set analysis aggregates data for complex traits based on biological data to reduce the sample size required to detect important signals. In this study, we used gene-set analysis of the GWAS for SNP-level associations with change in IBS-SSS to explore genome-mediated responses to treatment. To maximize power, we also combined participants from the three placebo treatment arms assuming placebo-related responses, which would contribute to the outcome in each of the three treatment arms. Four statistically significant GO terms were identified: three linked to transcription regulation (GO:0032968, GO:0070934, and GO:0070937) and one associated with DNA topoisomerase regulation (GO:0003918).

The genome-wide suggestive genetic loci plus *COMT* were densely connected in a transcription factor network in which *EGR1* was the transcription factor node with the greatest betweenness centrality. Gene expression analysis in this study demonstrated that *EGR1* was significantly downregulated from baseline after 6 weeks of the various forms of placebo treatment. *EGR1* is a critical mediator of gene-environment interactions and is tightly associated with neuronal activity and learning, memory, and sensitivity to reward. In rodents, water immersion restraint stress rapidly induces *EGR1* expression in blood vessels and gastroduodenal smooth muscle (29, 30). Similarly, *EGR1* expression is rapidly induced in jejunal smooth muscle and enteric neurons following surgical manipulation of the intestine, and *EGR1* expression in infiltrating mononuclear inflammatory cells correlates with postoperative ileus (31). Child abuse is associated with methylation of *EGR1* binding sites in the glucocorticoid receptor promoter region in PBMCs, thereby providing a mechanism by which social experience modulates hypothalamic–pituitary–adrenal axis activity (32). Similar epigenetic regulation by *EGR1* may be one of the mechanisms involved in IBS symptoms. As used in this study, the gene-set analysis provided potentially important insights into functional and biological mechanisms underlying the genetic component of placebo response.

Although the combined GWAS of all participants increased our power to detect loci associated with response to treatment in IBS, we were underpowered for a GWAS of the effects in the individual treatment arms, or stratified analyses by sex and IBS type (constipation or diarrhea). Despite the many known links of *COMT* to placebo and IBS, it did not emerge as a top hit in this GWAS. One possibility is that *COMT* effects are strongest with blinded-placebo, and the DBP arm in this study was underpowered for genome-wide significance. Another possibility is that the pharmacogenetic effects of *COMT*, which is known to interact with a wide variety of drugs and supplements, masked these effects (33–35). Although we were limited to PBMCs in this study to assess changes in gene expression, there is evidence that changes in PBMCs correlate with neurological changes in gene expression. Finally, in designing this trial, we expected that the NPC arm would serve as a control for “placebo effects.” However, with improvements among some IBS participants in the NPC, simply from enrolling in the trial, interacting with study staff, and responding to questionnaires at the study visits, we still cannot distinguish whether these effects are attributable to natural history or a modest placebo.

In the context of a randomized clinical trial largely consisting of placebo treatments, we have generalized the finding that *COMT* rs4680 genotype influences response to blinded placebo and used multi-omics analyses to acquire a more comprehensive view of the loci and pathways associated with treatment response in IBS. A deeper understanding of these pathways may guide the development of novel therapies for IBS (e.g., targeting *EGR1*) and improve the clinical trial design (e.g., excluding participants whose *COMT* genotype may predispose them to a significant placebo response).

## Data Availability Statement

The raw data supporting the conclusions of this article will be made available by the authors, without undue reservation.

## Ethics Statement

The studies involving human participants were reviewed and approved by Beth Israel Deaconess Medical Center under protocol 2015P000282. The patients/participants provided their written informed consent to participate in this study.

## Author Contributions

KH, AL, TK, JL, JS, and VC: project design, analysis, and writing manuscript. R-SW: analysis and writing manuscript. MR, JI, and JN: irritable bowel syndrome (IBS), trial design, execution, and writing manuscript. All authors contributed to the article and approved the submitted version.

## Funding

This study was supported, in part, by NIH grant R01AT008573 to AL and TK, NIH grants HL119145, HG007690, and GM107618, AHA grants D007382 and CV-19, and Rockefeller Foundation grant A015025 to JL, NIH K23 DK119584 to JS, and NIH K01HL130625 to KH, NIH K08 DK110532 to MR.

## Conflict of Interest

AL owns stock in Bristol Myer Squibb and Johnson & Johnson, and receives income from Vibrant and Mylan; JL is the scientific co-founder of Scipher Medicine, Inc. JN consults for World Clinical Care; MR receives research support from Boston Pharmaceuticals and Takeda for unrelated studies, and has consulted for 89Bio. MR's spouse is an employee of Takeda. JS has consulted for Takeda Pharmaceuticals and Teva Pharmaceuticals. The remaining authors declare that the research was conducted in the absence of any commercial or financial relationships that could be construed as a potential conflict of interest.

## Publisher's Note

All claims expressed in this article are solely those of the authors and do not necessarily represent those of their affiliated organizations, or those of the publisher, the editors and the reviewers. Any product that may be evaluated in this article, or claim that may be made by its manufacturer, is not guaranteed or endorsed by the publisher.

## Abbreviations

COMT: Catechol-O-methyltransferase
DBP: Double blind placebo
DBM: Double blind mint
IBS-SSS: IBS symptom severity scale
OLP: Open label placebo
NPC: No-pill control.

## References

[1] Lembo A Kelley JM Nee J Ballou S Iturrino J Cheng V. Open-label placebo vs. double-blind placebo for irritable bowel syndrome: a randomized clinical trial. Pain. (2021) 162:2428–35. 10.1097/j.pain.0000000000002234 33605656 PMC8357842

[2] Wager TD Atlas LY. The neuroscience of placebo effects: connecting context, learning and health. Nat Rev Neurosci. (2015) 16:403–18. 10.1038/nrn3976 26087681 PMC6013051

[3] Chen J Lipska BK Halim N Ma QD Matsumoto M Melhem S. Functional analysis of genetic variation in catechol-O-methyltransferase (COMT): effects on mRNA, protein, and enzyme activity in postmortem human brain. Am J Hum Genet. (2004) 75:807–21. 10.1086/425589 15457404 PMC1182110

[4] Lachman HM Papolos DF Saito T Yu YM Szumlanski CL Weinshilboum RM. Human catechol-O-methyltransferase pharmacogenetics: description of a functional polymorphism and its potential application to neuropsychiatric disorders. Pharmacogenetics. (1996) 6:243–50. 10.1097/00008571-199606000-00007 8807664

[5] Hall KT Lembo AJ Kirsch I Ziogas DC Douaiher J Jensen KB. Catechol-O-methyltransferase val158met polymorphism predicts placebo effect in irritable bowel syndrome. PLoS ONE. (2012) 7:e48135. 10.1371/journal.pone.0048135 23110189 PMC3479140

[6] Francis CY Morris J Whorwell PJ. The irritable bowel severity scoring system: a simple method of monitoring irritable bowel syndrome and its progress. Aliment Pharmacol Ther. (1997) 11:395–402. 10.1046/j.1365-2036.1997.142318000.x 9146781

[7] Kaptchuk TJ Hemond CC Miller FG. Placebos in chronic pain: evidence, theory, ethics, and use in clinical practice. BMJ. (2020) 370:m1668. 10.1136/bmj.m1668 32690477

[8] Rao S Lembo AJ Shiff SJ Lavins BJ Currie MG Jia XD. A 12-week, randomized, controlled trial with a 4-week randomized withdrawal period to evaluate the efficacy and safety of linaclotide in irritable bowel syndrome with constipation. Am J Gastroenterol. (2012) 107:1714–24; quiz p.1725. 10.1038/ajg.2012.255 22986440 PMC3504311

[9] Chey WD Lembo AJ Lavins BJ Shiff SJ Kurtz CB Currie MG. Linaclotide for irritable bowel syndrome with constipation: a 26-week, randomized, double-blind, placebo-controlled trial to evaluate efficacy and safety. Am J Gastroenterol. (2012) 107:1702–12. 10.1038/ajg.2012.254 22986437

[10] Purcell S Neale B Todd-Brown K Thomas L Ferreira MAR Bender D. PLINK: a tool set for whole-genome association and population-based linkage analyses. Am J Human Gene. (2007) 81:559–75. 17701901 10.1086/519795 PMC1950838

[11] Oni-Orisan A Haldar T Ranatunga DK Medina MW Schaefer C Krauss RM. The impact of adjusting for baseline in pharmacogenomic genome-wide association studies of quantitative change. NPJ Genom Med. (2020) 5:2–3. 10.1038/s41525-019-0109-4 31969989 PMC6965183

[12] de Leeuw CA Mooij JM Heskes T Posthuma D. MAGMA: generalized gene-set analysis of GWAS data. PLoS Comput Biol. (2015) 11:e1004219. 10.1371/journal.pcbi.1004219 25885710 PMC4401657

[13] Zhou G Soufan O Ewald J Hancock REW Basu N Xia J. NetworkAnalyst 3.0: a visual analytics platform for comprehensive gene expression profiling and meta-analysis. Nucleic Acids Res. (2019) 47:W234–41. 10.1093/nar/gkz240 30931480 PMC6602507

[14] Ballou S Kaptchuk TJ Hirsch W Nee J Iturrino J Hall KT. Open-label versus double-blind placebo treatment in irritable bowel syndrome: study protocol for a randomized controlled trial. Trials. (2017) 18:234. 10.1186/s13063-017-1964-x 28545508 PMC5445390

[15] Wang RS Croteau-Chonka DC Silverman EK Loscalzo J Weiss ST Hall KT. Pharmacogenomics and placebo response in a randomized clinical trial in asthma. Clin Pharmacol Ther. (2019) 106:1261–7. 10.1002/cpt.1646 31557306 PMC6851426

[16] McNeill EM Roos KP Moechars D Clagett-Dame M. Nav2 is necessary for cranial nerve development and blood pressure regulation. Neural Dev. (2010) 5:6. 10.1186/1749-8104-5-6 20184720 PMC2843687

[17] Davenport ER Cusanovich DA Michelini K Barreiro LB Ober C Gilad Y. Genome-wide association studies of the human gut microbiota. PLoS ONE. (2015) 10:e0140301. 10.1371/journal.pone.0140301 26528553 PMC4631601

[18] Dunbar E Greer PJ Melhem N Alkaade S Amann ST Brand R. Constant-severe pain in chronic pancreatitis is associated with genetic loci for major depression in the NAPS2 cohort. J Gastroenterol. (2020) 55:1000–9. 10.1007/s00535-020-01703-w 32681239 PMC9124361

[19] Nivard MG Mbarek H Hottenga JJ Smit JH Jansen R Penninx BW. Further confirmation of the association between anxiety and CTNND2: replication in humans. Genes Brain Behav. (2014) 13:195–201. 10.1111/gbb.12095 24256404

[20] Hughes DA Bacigalupe R Wang J Ruhlemann MC Tito RY Falony G. Genome-wide associations of human gut microbiome variation and implications for causal inference analyses. Nat Microbiol. (2020) 5:1079–87. 10.1038/s41564-020-0743-8 32572223 PMC7610462

[21] Rhee EP Ho JE Chen MH Shen D Cheng S Larson MG. A genome-wide association study of the human metabolome in a community-based cohort. Cell Metab. (2013) 18:130–43. 10.1016/j.cmet.2013.06.013 23823483 PMC3973158

[22] Fadista J Skotte L Geller F Bybjerg-Grauholm J Gortz S Romitti PA. Genome-wide meta-analysis identifies BARX1 and EML4-MTA3 as new loci associated with infantile hypertrophic pyloric stenosis. Hum Mol Genet. (2019) 28:332–40. 10.1093/hmg/ddy347 30281099 PMC6322072

[23] Ahuja R Pinyol R Reichenbach N Custer L Klingensmith J Kessels MM. Cordon-bleu is an actin nucleation factor and controls neuronal morphology. Cell. (2007) 131:337–50. 10.1016/j.cell.2007.08.030 17956734 PMC2507594

[24] Schumacher J Anthoni H Dahdouh F Konig IR Hillmer AM Kluck N. Strong genetic evidence of DCDC2 as a susceptibility gene for dyslexia. Am J Hum Genet. (2006) 78:52–62. 10.1086/498992 16385449 PMC1380223

[25] Zhang M Ergin V Lin L Stork C Chen L Zheng S. Axonogenesis is coordinated by neuron-specific alternative splicing programming and splicing regulator PTBP2. Neuron. (2019) 101:690–706 e10. 10.1016/j.neuron.2019.01.022 30733148 PMC6474845

[26] Kim W Zhao F Wu R Qin S Nowsheen S Huang J. ZFP161 regulates replication fork stability and maintenance of genomic stability by recruiting the ATR/ATRIP complex. Nat Commun. (2019) 10:5304. 10.1038/s41467-019-13321-z 31757956 PMC6876566

[27] Weidensdorfer D Stohr N Baude A Lederer M Kohn M Schierhorn A. Control of c-myc mRNA stability by IGF2BP1-associated cytoplasmic RNPs. RNA. (2009) 15:104–15. 10.1261/rna.1175909 19029303 PMC2612774

[28] Wang JC. DNA topoisomerases. Annu Rev Biochem. (1996) 65:635–92. 10.1146/annurev.bi.65.070196.003223 8811192

[29] Ueyama T Saika M Koreeda C Senba E. Water immersion-restraint stress induces expression of immediate-early genes in gastrointestinal tract of rats. Am J Physiol. (1998) 275:G287–95. 10.1152/ajpgi.1998.275.2.G287 9688656

[30] Senba E Ueyama T. Stress-induced expression of immediate early genes in the brain and peripheral organs of the rat. Neurosci Res. (1997) 29:183–207. 10.1016/S0168-0102(97)00095-3 9436645

[31] Schmidt J Stoffels B Moore BA Chanthaphavong RS Mazie AR Buchholz BM. Proinflammatory role of leukocyte-derived Egr-1 in the development of murine postoperative ileus. Gastroenterology. (2008) 135:926–36:936 e1–2. 10.1053/j.gastro.2008.05.079 18652830 PMC3319384

[32] Romens SE McDonald J Svaren J Pollak SD. Associations between early life stress and gene methylation in children. Child Dev. (2015) 86:303–9. 10.1111/cdev.12270 25056599 PMC4305348

[33] Hall KT Buring JE Mukamal KJ Vinayaga Moorthy M Wayne PM Kaptchuk TJ. COMT and alpha-tocopherol effects in cancer prevention: gene-supplement interactions in two randomized clinical trials. J Natl Cancer Inst. (2019) 111:684–94. 10.1093/jnci/djy204 30624689 PMC6624170

[34] Hall KT Kossowsky J Oberlander TF Kaptchuk TJ Saul JP Wyller VB. Genetic variation in catechol-O-methyltransferase modifies effects of clonidine treatment in chronic fatigue syndrome. Pharmacogenomics J. (2016) 16:454–60. 10.1038/tpj.2016.53 27457818 PMC5028250

[35] Hall KT Loscalzo J Kaptchuk TJ. Systems pharmacogenomics - gene, disease, drug and placebo interactions: a case study in COMT. Pharmacogenomics. (2019) 20:529–51. 10.2217/pgs-2019-0001 31124409 PMC6563236

