# Supplementary material for: Genomic Effects Associated With Response to Placebo Treatment in a Randomized Trial of Irritable Bowel Syndrome
Source: Front Pain Res (Lausanne). 2022 Jan 4;2:775386. doi: 10.3389/fpain.2021.775386 (PMC8915627; doi:10.3389/fpain.2021.775386)
Supplement: Supplementary file 1 [file Data_Sheet_1.docx]

Supplementary Material


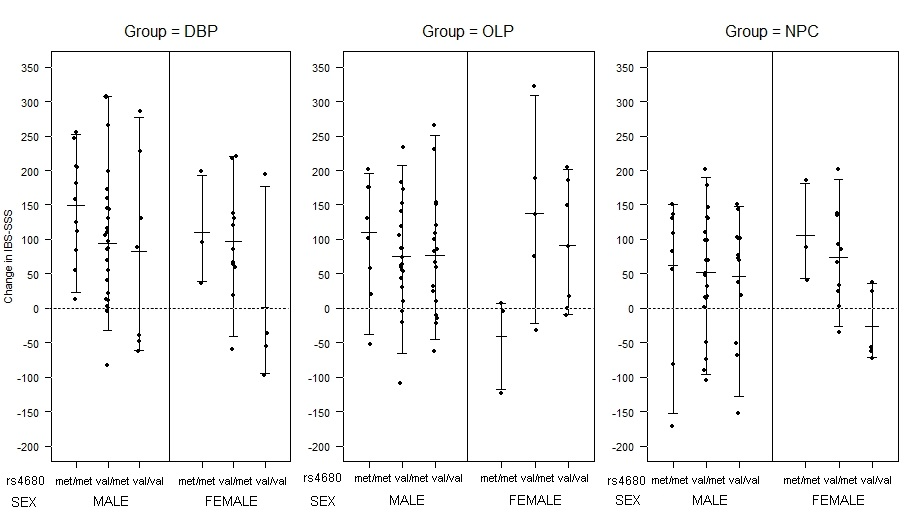


Female Male

Female Male

Female Male

**Supplementary Figure 1**. Change in IBS-SSS in each treatment arm (DBP = double-blind, OLP = open-label and NPC = no-pill control) by sex and COMT rs4680 genotype


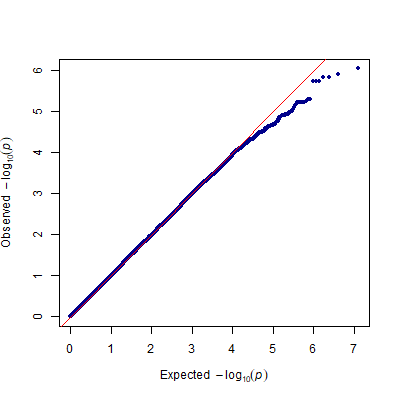


**Supplementary Figure 2.** QQ plot of GWAS of change in IBS-SSS Lambda=0.986

| **Supplementary Table 1.** Results of MAGMA gene-set analysis showing genetic loci for the four genome-wide significant gene-sets. number of tests = 15488; P-value for significance < 3.22E-06   \| **GO_bp:go_regulation_of_transcription_elongation_from_rna_polymerase_ii_promoter (set) NGENES = 29 P-VALUE = 1.23E-06** \| \| --- \| \| \| \| | | | | | | | | | | | |
| --- | --- | --- | --- | --- | --- | --- | --- | --- | --- | --- | --- | --- |
|  |  |  |  |  |  |  |  |  |  |  |  |
|  |  |  |  |  |  |  |  |  |  |  |  |
|  |  |  |  |  |  |  |  |  |  |  |  |
| Set 1 | GENE | CHR | START | STOP | NSNPS | NPARAM | N | ZSTAT | P | ZFITTED_BASE | ZRESID_BASE |
|  | ENSG00000134371 | 1 | 193091147 | 193223031 | 98 | 3 | 188 | -0.87554 | 0.8038 | 0 | -0.87554 |
|  | ENSG00000153187 | 1 | 245014468 | 245027844 | 8 | 2 | 188 | 1.1665 | 0.12045 | 0 | 1.1665 |
|  | ENSG00000163961 | 3 | 196195654 | 196230639 | 89 | 6 | 188 | 1.0833 | 0.13717 | 0 | 1.0833 |
|  | ENSG00000185049 | 4 | 1984441 | 2043630 | 82 | 7 | 188 | -0.33309 | 0.62698 | 0 | -0.33309 |
|  | ENSG00000204356 | 6 | 31919864 | 31926887 | 11 | 3 | 188 | 0.71162 | 0.23953 | 0 | 0.71162 |
|  | ENSG00000112130 | 6 | 37321748 | 37362514 | 21 | 5 | 188 | 1.919 | 0.027066 | 0 | 1.919 |
|  | ENSG00000065883 | 7 | 39989636 | 40136733 | 218 | 11 | 188 | 0.61977 | 0.26675 | 0 | 0.61977 |
|  | ENSG00000106462 | 7 | 148504475 | 148581413 | 115 | 11 | 188 | -0.51819 | 0.69501 | 0 | -0.51819 |
|  | ENSG00000164690 | 7 | 155592680 | 155604967 | 18 | 7 | 188 | 2.6349 | 0.0042402 | 0 | 2.6349 |
|  | ENSG00000154582 | 8 | 74851404 | 74884522 | 62 | 6 | 188 | -0.021156 | 0.50392 | 1.04E-17 | -0.021156 |
|  | ENSG00000136807 | 9 | 130547958 | 130553066 | 6 | 1 | 188 | -0.59051 | 0.7153 | 0 | -0.59051 |
|  | ENSG00000188986 | 9 | 140149625 | 140167998 | 49 | 2 | 188 | -0.24641 | 0.58865 | 0 | -0.24641 |
|  | ENSG00000015171 | 10 | 180405 | 300577 | 109 | 7 | 188 | -0.45639 | 0.6719 | 0 | -0.45639 |
|  | ENSG00000198730 | 11 | 10772534 | 10801290 | 28 | 6 | 188 | 0.77551 | 0.2178 | 0 | 0.77551 |
|  | ENSG00000233436 | 11 | 57510986 | 57519253 | 1 | 1 | 188 | -0.42144 | 0.662 | 0 | -0.42144 |
|  | ENSG00000188342 | 13 | 45694650 | 45858237 | 187 | 3 | 188 | -1.4011 | 0.91768 | 0 | -1.4011 |
|  | ENSG00000092201 | 14 | 21819631 | 21852425 | 41 | 3 | 188 | 1.4211 | 0.077293 | 0 | 1.4211 |
|  | ENSG00000129518 | 14 | 34985135 | 35008916 | 67 | 7 | 188 | -1.0229 | 0.84443 | 0 | -1.0229 |
|  | ENSG00000137815 | 15 | 41700606 | 41775761 | 64 | 4 | 188 | 0.19918 | 0.41491 | 0 | 0.19918 |
|  | ENSG00000166477 | 15 | 52230222 | 52264003 | 56 | 2 | 188 | 0.1926 | 0.41288 | 0 | 0.1926 |
|  | ENSG00000140395 | 15 | 78570177 | 78592136 | 43 | 9 | 188 | -0.11644 | 0.5442 | 1.39E-17 | -0.11644 |
|  | ENSG00000109111 | 17 | 26989109 | 27029697 | 22 | 3 | 188 | 0.81915 | 0.20546 | 0 | 0.81915 |
|  | ENSG00000167258 | 17 | 37617764 | 37721160 | 135 | 4 | 188 | 0.13967 | 0.43983 | 0 | 0.13967 |
|  | ENSG00000213246 | 17 | 56422539 | 56430454 | 19 | 4 | 188 | 0.30428 | 0.37694 | 0 | 0.30428 |
|  | ENSG00000108469 | 17 | 73622925 | 73663269 | 34 | 3 | 188 | 2.5845 | 0.0046406 | 0 | 2.5845 |
|  | ENSG00000125651 | 19 | 6379580 | 6393992 | 20 | 3 | 188 | -0.63897 | 0.73926 | 0 | -0.63897 |
|  | ENSG00000141867 | 19 | 15347647 | 15443356 | 59 | 4 | 188 | -0.41586 | 0.65624 | 0 | -0.41586 |
|  | ENSG00000105656 | 19 | 18553473 | 18632937 | 156 | 7 | 188 | 0.74697 | 0.22552 | 0 | 0.74697 |
|  | ENSG00000006712 | 19 | 39876492 | 39881835 | 2 | 1 | 188 | 1.1596 | 0.12497 | 0 | 1.1596 |

**GO_bp:go_crd_mediated_mrna_stabilization (set)**

**NGENES = 5**

**P-VALUE = 7.14E-08**

| Set 2 | GENE | CHR | START | STOP | NSNPS | NPARAM | N | ZSTAT | P | ZFITTED_BASE | ZRESID_BASE |
| --- | --- | --- | --- | --- | --- | --- | --- | --- | --- | --- | --- |
|  | ENSG00000065978 | 1 | 43148098 | 43168020 | 17 | 4 | 188 | 0.68997 | 0.24282 | 0 | 0.68997 |
|  | ENSG00000135829 | 1 | 182808504 | 182856886 | 34 | 3 | 188 | -1.0537 | 0.85468 | 0 | -1.0537 |
|  | ENSG00000153187 | 1 | 245014468 | 245027844 | 8 | 2 | 188 | 1.1665 | 0.12045 | 0 | 1.1665 |
|  | ENSG00000135316 | 6 | 86318053 | 86353510 | 37 | 5 | 188 | 0.31785 | 0.37195 | 0 | 0.31785 |
|  | ENSG00000159217 | 17 | 47074774 | 47133012 | 71 | 7 | 188 | 1.2967 | 0.095369 | 0 | 1.2967 |

| **GO_bp:go_regulation_of_dna_topoisomerase_atp_hydrolyzing_activity (set) NGENES = 5 P-VALUE = 3.06E-06** |
| --- |
|  |
|  |

| Set 3 | GENE | CHR | START | STOP | NSNPS | NPARAM | N | ZSTAT | P | ZFITTED_BASE | ZRESID_BASE |
| --- | --- | --- | --- | --- | --- | --- | --- | --- | --- | --- | --- |
|  | ENSG00000135829 | 1 | 182808504 | 182856886 | 34 | 3 | 188 | -1.0537 | 0.85468 | 0 | -1.0537 |
|  | ENSG00000153187 | 1 | 245014468 | 245027844 | 8 | 2 | 188 | 1.1665 | 0.12045 | 0 | 1.1665 |
|  | ENSG00000121152 | 2 | 97001525 | 97039583 | 26 | 3 | 188 | -1.2972 | 0.90012 | 0 | -1.2972 |
|  | ENSG00000170364 | 3 | 4344988 | 4359251 | 24 | 6 | 188 | 1.2065 | 0.11293 | 0 | 1.2065 |
|  | ENSG00000188313 | 3 | 146232967 | 146262651 | 113 | 10 | 188 | 0.99214 | 0.1589 | 0 | 0.99214 |

| **GO_cc:go_crd_mediated_mrna_stability_complex (set) NGENES = 6 P-VALUE = 1.66E-07** |
| --- |
|  |
|  |

| Set 4 | GENE | CHR | START | STOP | NSNPS | NPARAM | N | ZSTAT | P | ZFITTED_BASE | ZRESID_BASE |
| --- | --- | --- | --- | --- | --- | --- | --- | --- | --- | --- | --- |
|  | ENSG00000065978 | 1 | 43148098 | 43168020 | 17 | 4 | 188 | 0.68997 | 0.24282 | 0 | 0.68997 |
|  | ENSG00000009307 | 1 | 115259534 | 115301297 | 49 | 4 | 188 | 1.0469 | 0.14475 | 0 | 1.0469 |
|  | ENSG00000135829 | 1 | 182808504 | 182856886 | 34 | 3 | 188 | -1.0537 | 0.85468 | 0 | -1.0537 |
|  | ENSG00000153187 | 1 | 245014468 | 245027844 | 8 | 2 | 188 | 1.1665 | 0.12045 | 0 | 1.1665 |
|  | ENSG00000135316 | 6 | 86318053 | 86353510 | 37 | 5 | 188 | 0.31785 | 0.37195 | 0 | 0.31785 |
|  | ENSG00000159217 | 17 | 47074774 | 47133012 | 71 | 7 | 188 | 1.2967 | 0.095369 | 0 | 1.2967 |

**Supplementary Table 2.** Seed and transcription factor network from Network Analyst showing EGR1 as the transcription factor with the highest degree and betweeness centrality

| ID | Label | Degree | Betweenness |
| --- | --- | --- | --- |
| 89797 | NAV2 | 55 | 3072 |
| 1501 | CTNND2 | 42 | 1775.96 |
| 152579 | SCFD2 | 35 | 1659.4 |
| 1312 | COMT | 29 | 1458.52 |
| 58155 | PTBP2 | 29 | 1062.96 |
| 7541 | ZBTB14 | 25 | 1339.29 |
| 51473 | DCDC2 | 19 | 548.23 |
| 83643 | CCDC3 | 19 | 478.24 |
| **1958** | **EGR1** | **7** | **406.97** |
| 7157 | TP53 | 7 | 297.74 |
| 195977 | ANTXRL | 7 | 147.57 |
| 1390 | CREM | 6 | 289.81 |
| 861 | RUNX1 | 6 | 251.91 |
| 3172 | HNF4A | 6 | 229.32 |
| 3170 | FOXA2 | 5 | 240.15 |
| 6929 | TCF3 | 5 | 194.77 |
| 367 | AR | 5 | 173.67 |
| 4286 | MITF | 5 | 171.34 |
| 6657 | SOX2 | 5 | 140.33 |
| 2313 | FLI1 | 5 | 115.25 |
| 4089 | SMAD4 | 5 | 95.29 |
| 6688 | SPI1 | 4 | 164.92 |
| 2623 | GATA1 | 4 | 122.92 |
| 6774 | STAT3 | 4 | 91.16 |
| 2099 | ESR1 | 4 | 90.83 |
| 6662 | SOX9 | 4 | 77.72 |
| 6925 | TCF4 | 4 | 77.58 |
| 1385 | CREB1 | 4 | 73.5 |
| 10413 | YAP1 | 4 | 58.69 |
| 22823 | MTF2 | 4 | 57.34 |
| 23512 | SUZ12 | 4 | 57.34 |
| 1869 | E2F1 | 3 | 94.31 |
| 6886 | TAL1 | 3 | 94.31 |
| 57167 | SALL4 | 3 | 67.07 |
| 5468 | PPARG | 3 | 65.06 |
| 6597 | SMARCA4 | 3 | 46.88 |
| 7022 | TFAP2C | 3 | 43.21 |
| 5077 | PAX3 | 3 | 40.17 |
| 5460 | POU5F1 | 3 | 37.24 |
| 4609 | MYC | 3 | 35.25 |
| 7764 | ZNF217 | 3 | 33.97 |
| 2002 | ELK1 | 3 | 33.49 |
| 7004 | TEAD4 | 3 | 30.63 |
| 8626 | TP63 | 3 | 29.84 |
| 79923 | NANOG | 3 | 29.62 |
| 2100 | ESR2 | 3 | 29.62 |
| 5467 | PPARD | 3 | 23 |
| 5970 | RELA | 2 | 43.98 |
| 10661 | KLF1 | 2 | 28.95 |
| 5927 | KDM5A | 2 | 25.37 |
| 10215 | OLIG2 | 2 | 25.37 |
| 1523 | CUX1 | 2 | 25.37 |
| 2908 | NR3C1 | 2 | 23.05 |
| 2103 | ESRRB | 2 | 22.61 |
| 23309 | SIN3B | 2 | 21.38 |
| 9869 | SETDB1 | 2 | 21.38 |
| 4211 | MEIS1 | 2 | 21.38 |
| 2624 | GATA2 | 2 | 18.21 |
| 2626 | GATA4 | 2 | 18.21 |
| 5087 | PBX1 | 2 | 17.4 |
| 3725 | JUN | 2 | 15.85 |
| 8856 | NR1I2 | 2 | 15.85 |
| 23528 | ZNF281 | 2 | 15.71 |
| 1874 | E2F4 | 2 | 14.68 |
| 2146 | EZH2 | 2 | 14.05 |
| 7543 | ZFX | 2 | 13.38 |
| 152118 | C3ORF79 | 2 | 13.03 |
| 5978 | REST | 2 | 12.06 |
| 51540 | SCLY | 2 | 12.06 |
| 571 | BACH1 | 2 | 12.06 |
| 10155 | TRIM28 | 2 | 11.34 |
| 63978 | PRDM14 | 2 | 9.95 |
| 1052 | CEBPD | 2 | 7.22 |
| 860 | RUNX2 | 2 | 7.22 |
| 3720 | JARID2 | 2 | 7.22 |
| 23186 | RCOR1 | 2 | 7.22 |
| 2130 | EWSR1 | 2 | 7.22 |
| 29989 | OBP2B | 2 | 0.81 |
| 405 | ARNT | 1 | 0 |
| 4780 | NFE2L2 | 1 | 0 |
| 2113 | ETS1 | 1 | 0 |
| 3225 | HOXC9 | 1 | 0 |
| 1602 | DACH1 | 1 | 0 |
| 23133 | PHF8 | 1 | 0 |
| 9314 | KLF4 | 1 | 0 |
| 10765 | KDM5B | 1 | 0 |
| 55758 | RCOR3 | 1 | 0 |
| 27086 | FOXP1 | 1 | 0 |
| 80312 | TET1 | 1 | 0 |
| 2078 | ERG | 1 | 0 |
| 8328 | GFI1B | 1 | 0 |
| 4849 | CNOT3 | 1 | 0 |
| 6736 | SRY | 1 | 0 |
| 2033 | EP300 | 1 | 0 |
| 7490 | WT1 | 1 | 0 |
| 1761 | DMRT1 | 1 | 0 |
| 29842 | TFCP2L1 | 1 | 0 |
| 467 | ATF3 | 1 | 0 |
| 4087 | SMAD2 | 1 | 0 |
| 4088 | SMAD3 | 1 | 0 |
| 1105 | CHD1 | 1 | 0 |
| 7528 | YY1 | 1 | 0 |
| 132625 | ZFP42 | 1 | 0 |
| 4205 | MEF2A | 1 | 0 |
| 6722 | SRF | 1 | 0 |
| 6910 | TBX5 | 1 | 0 |
| 6932 | TCF7 | 1 | 0 |
| 5885 | RAD21 | 1 | 0 |
| 648 | BMI1 | 1 | 0 |
| 8726 | EED | 1 | 0 |
| 6045 | RNF2 | 1 | 0 |
| 7020 | TFAP2A | 1 | 0 |
| 1499 | CTNNB1 | 1 | 0 |
| 190 | NR0B1 | 1 | 0 |
| 1045 | CDX2 | 1 | 0 |
| 6721 | SREBF2 | 1 | 0 |
| 11107 | PRDM5 | 1 | 0 |
| 23569 | PADI4 | 1 | 0 |
| 3516 | RBPJ | 1 | 0 |
| 6772 | STAT1 | 1 | 0 |
| 27000 | DNAJC2 | 1 | 0 |
| 1406 | CRX | 1 | 0 |
| 9575 | CLOCK | 1 | 0 |
| 283248 | RCOR2 | 1 | 0 |
| 2308 | FOXO1 | 1 | 0 |
| 29102 | DROSHA | 1 | 0 |
| 2637 | GBX2 | 1 | 0 |
| 2305 | FOXM1 | 1 | 0 |
| 63976 | PRDM16 | 1 | 0 |
