# Supplementary material for: Genomic Effects Associated With Response to Placebo Treatment in a Randomized Trial of Irritable Bowel Syndrome
Source: Front Pain Res (Lausanne). 2022 Jan 4;2:775386. doi: 10.3389/fpain.2021.775386 (PMC8915627; doi:10.3389/fpain.2021.775386)
Supplement: Supplementary file 2 [file Data_Sheet_2.docx]

Supplementary methods.

**Genotyping**

Genotyped was conducted on the Infinium Global Screening Array v2.0 (Illumina, San Diego, Calif). All samples were available for QC analyses with 734,288 single nucleotide variant (SNVs). After filtering the extremely low-quality samples and variants (call rate < 97.5%), 730,526 SNVs were left for further QC analyses using PLINK (version 1.9) ,[1]. After 1,510 SNVs were excluded from the dataset due to their not being in Hardy-Weinberg equilibrium, 729,016 SNVs remained. Twelve samples were removed (1 for excess heterozygosity, 5 samples with gender mismatch, 1 pair with strong familial relationships, and 4 with label errors). SNPs were mapped to the GRCh37 (hg19) reference genome. To reduce heterogeneity in population structure, we used PLINK to perform principal component analysis (PCA) on the whole genome SNP data and extracted the top 5 principal components for correcting genetic heterogeneity across different races/ethnic groups.

Imputation was performed using a two-step procedure --- Eagle phasing and Minimac imputation for all the subjects ,[2]. The number of SNPs imputed was 48,935,166, which is the expected number after using the 1000GP3v5 reference panel. The genome coordinates build involved use of GRCh37 ,[3]. As quality control steps for conducting the GWAS, we used PLINK to extract SNPs with minor allele frequency (MAF) >0.05 and with a Hardy-Weinberg equilibrium (HWE) P>10^-6^.

**Gene expression**

A candidate gene expression analysis for *EGR1* was conducted in this study. A detailed report of our gene expression findings will be published elsewhere. RNA-seq (Differential Gene Expression Analysis) was performed at Admera Health, Plainfield NJ. Briefly, RNA was extracted from human blood using PAXgene Blood RNA kit (Qiagen, Hilden, Germany) at baseline and six-weeks. Isolated RNA sample quality was assessed by BioAnalyzer RNA 6000 pico (Agilent Technologies Inc., California, USA) and quantified by Qubit 2.0 RNA HS assay (ThermoFisher, Massachusetts, USA). Qiaseq FastSelect RNA removal kit (Qiagen, Hilden, Germany) was used to deplete ribosomal RNA. Prior to first strand synthesis, samples were randomly primed (5´ d(N6) 3´ [N=A,C,G,T]) and fragmented based on the manufacturer’s recommendations. The first strand was synthesized with a longer extension period, 30 minutes at 42⁰C, and all remaining steps for library construction were used according to the NEBNext® Ultra™ II Non-Directional RNA Library Prep Kit for Illumina® (New England BioLabs Inc., Massachusetts, USA). Final libraries were assessed by Qubit 2.0 (ThermoFisher, Massachusetts, USA) and quality was assessed by TapeStation D1000 ScreenTape (Agilent Technologies Inc., California, USA). Final library size was approximately 400bp with an insert size of about 250bp. Illumina® 8-nt dual-indices were used. Equimolar pooling of libraries was performed based on QC values and sequenced on an Illumina® NovaSeq S4 (Illumina, California, USA) with a read length configuration of 150 PE for 60 M PE reads per sample (30M in each direction). FastQC v0.11.8 ,[4] was used to check the quality of raw reads. Trimmomatic (version 0.38) ,[5] was used to cut adaptors and remove low quality bases. The clean reads were then mapped onto hg19 using bowtie2 ,[6]. Htseq-count version 0.11.2 was used to count the number of mapped reads in genes ,[7]. R package edgeR was used to normalize the raw count data on the basis of library complexity and gene variation ,[8]. The differentially expressed *EGR1* was identified on the basis of fold change. These values were used for subsequent differential expression analysis using a pairwise t test.

References

[1] S. Purcell, B. Neale, K. Todd-Brown, L. Thomas, M.A. Ferreira, D. Bender, J. Maller, P. Sklar, P.I. de Bakker, M.J. Daly, and P.C. Sham, PLINK: a tool set for whole-genome association and population-based linkage analyses. Am J Hum Genet 81 (2007) 559-75.

[2] R.J. Eller, S.C. Janga, and S. Walsh, Odyssey: a semi-automated pipeline for phasing, imputation, and analysis of genome-wide genetic data. BMC Bioinformatics 20 (2019) 364.

[3] S. Das, L. Forer, S. Schonherr, C. Sidore, A.E. Locke, A. Kwong, S.I. Vrieze, E.Y. Chew, S. Levy, M. McGue, D. Schlessinger, D. Stambolian, P.R. Loh, W.G. Iacono, A. Swaroop, L.J. Scott, F. Cucca, F. Kronenberg, M. Boehnke, G.R. Abecasis, and C. Fuchsberger, Next-generation genotype imputation service and methods. Nat Genet 48 (2016) 1284-1287.

[4] S. Andrews, A Quality Control Tool for High Throughput Sequence Data 2010.

[5] A.M. Bolger, M. Lohse, and B. Usadel, Trimmomatic: a flexible trimmer for Illumina sequence data. Bioinformatics 30 (2014) 2114-20.

[6] B. Langmead, and S.L. Salzberg, Fast gapped-read alignment with Bowtie 2. Nat Methods 9 (2012) 357-9.

[7] S. Anders, P.T. Pyl, and W. Huber, HTSeq--a Python framework to work with high-throughput sequencing data. Bioinformatics 31 (2015) 166-9.

[8] M.D. Robinson, D.J. McCarthy, and G.K. Smyth, edgeR: a Bioconductor package for differential expression analysis of digital gene expression data. Bioinformatics 26 (2010) 139-40.
